# Intraspecific Variation in Microsatellite Mutation Profiles in *Daphnia magna*

**DOI:** 10.1093/molbev/msz118

**Published:** 2019-05-11

**Authors:** Eddie K H Ho, Fenner Macrae, Leigh C Latta, Maia J Benner, Cheng Sun, Dieter Ebert, Sarah Schaack

**Affiliations:** 1 Department of Biology, Reed College, Portland, OR; 2 Institute of Apicultural Research, Chinese Academy of Agricultural Sciences, Beijing, China; 3 Department of Environmental Sciences, Zoology, University of Basel, Basel, Switzerland; 4 Division of Natural Sciences and Mathematics, Lewis-Clark State College, Lewiston, ID

**Keywords:** tandem repeats, mutation rate variation, mutation accumulation, waterfleas, Cladocera

## Abstract

Microsatellite loci (tandem repeats of short nucleotide motifs) are highly abundant in eukaryotic genomes and often used as genetic markers because they can exhibit variation both within and between populations. Although widely recognized for their mutability and utility, the mutation rates of microsatellites have only been empirically estimated in a few species, and have rarely been compared across genotypes and populations within a species. Here, we investigate the dynamics of microsatellite mutation over long- and short-time periods by quantifying the starting abundance and mutation rates for microsatellites for six different genotypes of *Daphnia magna*, an aquatic microcrustacean, collected from three populations (Finland, Germany, and Israel). Using whole-genome sequences of these six starting genotypes, descendent mutation accumulation (MA) lines, and large population controls (non-MA lines), we find each genotype exhibits a distinctive initial microsatellite profile which clusters according to the population-of-origin. During the period of MA, we observe motif-specific, highly variable, and rapid microsatellite mutation rates across genotypes of *D. magna*, the average of which is order of magnitude greater than the recently reported rate observed in a single genotype of the congener, *Daphnia pulex*. In our experiment, genotypes with more microsatellites starting out exhibit greater losses and those with fewer microsatellites starting out exhibit greater gains—a context-dependent mutation bias that has not been reported previously. We discuss how genotype-specific mutation rates and spectra, in conjunction with evolutionary forces, can shape both the differential accumulation of repeat content in the genome and the evolution of mutation rates.

## Introduction

Microsatellite loci, also known as short tandem repeats, are repetitive regions of the genome known for their propensity to mutate rapidly (e.g., Sun et al. 2012). Although exact definitions of microsatellites vary, they typically involve tandem arrays of short motifs (typically, 1–6 bp long, although longer motifs can also be found in tandem arrays). Microsatellites can be located inside or outside coding regions of the genome, and have been shown to influence a range of phenotypes from gene expression to genetic disease (Feupe Fotsing et al. 2018). Previous reports of microsatellite mutation rates have consistently shown them to be higher than substitution rates in unique sequence, often by several orders of magnitude (reviewed by Ellegren 2004). Because of their mutability, microsatellites have frequently been used in population genetics studies and there is increasing interest in the role they may play in adaptation, plasticity, and disease (Haasl and Payseur 2013; Hannan 2018).

There are three mechanisms of mutation that have been proposed to explain the patterns of higher mutation rates at microsatellite loci: retrotransposition, unequal crossing over, and DNA slippage. Retrotransposition, in particular, could explain the frequent observation that microsatellites tend to be A-rich, although it is less clear how retrotransposition would impact mutation rates of microsatellites once they are formed. Unequal crossing over is thought to increase in frequency at repeat-rich loci and can, potentially, lead to the expansion or contraction of tandem arrays with equal probability. The most often discussed mechanism of microsatellite mutation is strand slippage during DNA replication and repair (Kornberg et al. 1964), whereby the array of repeats can cause potential mispairing between template and nascent strands of DNA. If uncorrected by DNA repair mechanisms, slippage can lead to the expansion or contraction of a tandem array and may do so in a motif- or length-dependent manner (Eckert and Hile 2009). When substitutions occur at microsatellite repeats, they result in the loss (or “death”) of the repeat, in addition to loss or contraction due to deletions or contractions during slippage (Kelkar et al. 2011). A given microsatellite locus can experience any of a number of different types of mutation (e.g., insertions, deletions, duplications, slippage, and substitutions) which can result in either an expansion or contraction of that tandem array, the interruption of the array, or the increase or decrease in copy number of the array. Because all these mutation types will contribute to overall copy number for any given motif (referred to as a k-mer, hereafter), genome-wide analyses of microsatellite mutation rates can benefit from looking at rates of copy number increase and decrease as a global metric of the impact of mutation at these loci.

Microsatellite mutation rate variation based on the composition of the motif (AT-/GC-content), length of the motif (unit length; e.g., dinucleotide vs. trinucleotide repeats), and the length of the array (e.g., the number of repeats occurring in tandem at a given locus) has been the focus of previous studies in a variety of systems (reviewed by Bhargava and Fuentes 2010). Theoretically, mutation rates would be expected (1) to be higher in AT-rich regions (due to the lower number of hydrogen bonds between base pairs), (2) to decrease as a function of unit length based on the strand slippage model of mutation, and (3) to increase as a function of array length, given the increased number of targets for mutation (reviewed by Eckert and Hile 2009). Indeed, empirical studies have shown that microsatellites with high AT-content tend to mutate at higher rates than those that are GC-rich and that dinucleotide rates are higher than trinucleotide repeats (Chakraborty et al. 1997). Rates of expansion versus contraction, however, have been shown to depend on starting length, with shorter arrays tending to increase in length and longer arrays tending to decrease in length (Lai and Sun 2003; Seyfert et al. 2008). Indeed, if microsatellite mutation rates vary based on any of these factors, one could make predictions about the accumulation of microsatellites across the genome over long time periods based on starting composition of the repeat content.

As with most types of mutations, mutation rate estimates are typically performed on one or a few genotypes for a representative model species, and then used to extrapolate mutation rate estimates for congeners, or even more widely, despite a lack of evidence for generalizing to this degree (e.g., mutation rate estimates for *Drosophila melanogaster* are routinely used as a proxy for all insects, despite known variation in rates estimates between genotypes [Haag-Liautard et al. 2007]). The degree to which microsatellite mutation rates and patterns of microsatellite accumulation vary between genotypes and populations, intraspecifically, or among closely-related species with similar lifespans, physiologies, and life histories has remained largely unexplored. Given that the rate of mutation itself is a trait that can evolve, knowing the level of intraspecific variation upon which evolutionary forces can act to increase or decrease the rate over time (Lynch 2010), as well as knowing what factors influence rate differences, is of major interest to biologists (Baer et al. 2007). Mutation accumulation (MA) experiments provide the least biased estimates of mutation rates available (Halligan and Keightley 2008), although they can only be conducted in organisms that can be reared in a controlled environment with short generation times. The basis of MA experiments is to compare the number of accumulated mutations in lineages propagated via single-progeny descent (a genetic bottleneck that minimizes natural selection and maximizes genetic drift) to the starting genotype or to the genotype of lineages maintained in parallel at large population sizes where selection is not minimized. *Daphnia*, in particular, are well-suited to MA experiments because of their ability to reproduce asexually (thereby eliminating the loss of any mutations during gametogenesis), their short generation time, and the long history of research using the system (Schaack 2008; Miner et al. 2012).

Here, we examine microsatellite mutation rates in three *Daphnia magna* populations, and compare our results to those obtained for *Daphnia pulex* (Flynn et al. 2017). We characterize both the microsatellite landscape and mutational profiles from six genotypes of *D. magna*—two each from three populations (Finland, Germany, and Israel), in order to determine if there is a relationship between the two, and to assess the degree to which they may vary among genotypes, populations, and closely-related species. In addition, we report the microsatellites that are most abundant and most mutable, to determine if there are features of individual microsatellites (unit length or content) that determine differences in mutation dynamics among loci. Identifying patterns using MA data collected on experimental time-scales where selection is minimized and contrasting such data with patterns of microsatellite accumulation over long time periods can reveal the degree to which evolutionary forces are shaping microsatellite landscapes in nature.

## Results

### Microsatellite Copy Number Profiles in *D. magna*

We scanned for microsatellite content from sequenced reads using *k-Seek* (Wei et al. 2014) which detects motifs with lengths 1–20 bp (k-mers) repeating tandemly for at least 50 bp on a given read, allowing for 1 bp mismatch per repeat unit. The program *k-Seek* outputs the total count of a given k-mer across all locations in the genome that satisfies these requirements. Due to this threshold, *k-Seek* excludes all microsatellite loci that span less than 50 bp, resulting in an underestimate of total repeat content and a detection bias such that repeats containing shorter k-mers must have a higher number of units to be included in any downstream analyses. By modifying the parameters of *k-Seek* to include all microsatellite loci spanning at least 10 bp (see Supplementary Material online), we confirmed that using the default higher minimum threshold does mean we are consistently underestimating the total microsatellite content in the genome (supplementary table S8, Supplementary Material online), but that estimates of mutation rates are not biased by this subsampling (supplementary fig. S9, Supplementary Material online). For the results presented here, we use the default *k-Seek* threshold of 50 bp so that our results are more directly comparable to other studies that have estimated genome-wide microsatellite copy number and mutation rates using *k-Seek* (Wei et al. 2014; Flynn et al. 2017, 2018) and those that will in the future.

We scanned for motifs (k-mers) of lengths up to 20 bp across the genome of individuals sequenced from 47 MA lines derived from 6 starting genotypes (“starting controls” [SC]) and 12 lines (2 per starting genotype) maintained in large population sizes in parallel with the MA lines sampled at the end of the experimental period (“extant controls” [EC]). After normalization by depth of coverage, the total number of base pairs (per 1× coverage) composed of k-mers ranged from 97 to 145 Kb across our six *D. magna* starting genotypes, which represented 0.07% to 0.1% of the 141 Mb *D. magna* reference genome (supplementary fig. S2, Supplementary Material online). Across all SC, EC and MA lines, the median number of base pairs composed of k-mers was 121 kb (0.085% of the genome). In contrast, the median k-mer content of *D. pulex* was 1.2 MB (0.6% of the estimated 200 Mb *D. pulex* genome), which is an order of magnitude higher than in *D. magna* (Flynn et al. 2017). The k-mer content in our *D. magna* lines was more similar to that in *Chlamydomonas reinhardtii*, which contains an average of 180 Kb (0.15% of the genome) (Flynn et al. 2018).

**Fig. 1.**
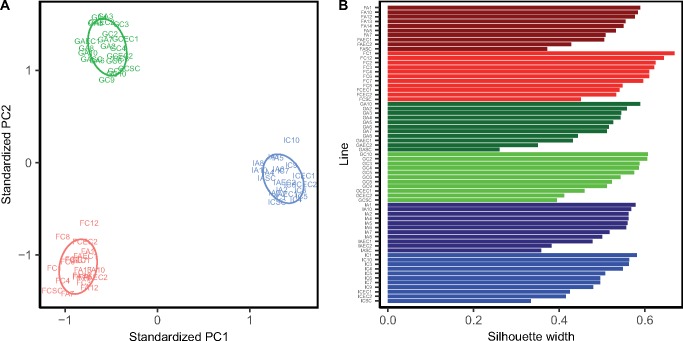
Population structure using the 100 k-mers with nonzero copy number across all 65 lines. (*A*) Each line is plotted based on the first and second principle components axis. Lines from Finland, Germany, and Israel are colored red, green and blue, respectively. (*B*) k-medoids analysis using the first ten PCs of the principal components analysis. Each cluster only contained one starting genotype (SC) and all of its descendant MA and EC lines. Dark red, red, dark green, green, dark blue, and blue represent lines from genotypes FA, FC, GA, GC, IA, IC, respectively.

We performed a principal components analysis (PCA) using the copy number of the 100 k-mers (average repeat unit length of 10.9) with nonzero copy numbers across all 65 lines in order to look for distinctive patterns of the microsatellite landscape across the 6 genotypes. On the first and second principle components axes, the lines clearly clustered based on their population-of-origin (fig. 1). We additionally performed a k-medoids analysis using the first ten principle components (these ten PCs explained 83% of the variation in copy number). We found that six clusters maximized the average silhouette across lines. Each of these six clusters contained the SC line, all of its descendent MA lines, and the EC of that genotype (fig. 1). Overall, the k-mer copy number profiles distinguished lines based on their population and genotype.

Our PCA results are conservative because we only examine k-mers shared across all lines (including the k-mers unique to each population would only increase the degree of clustering observed). We observed 92, 91, and 127 k-mers, respectively, that only exist in the lines from Finland, Germany, and Israel. Unsurprisingly, the average repeat unit length of these population-specific k-mers were 13.8, 14.1, and 12.8 for Finland, Germany, and Israel, respectively, which are larger than the average of the 100 shared k-mers. The vast majority of population-specific k-mers are low in abundance, with an average copy number below 25. The exceptions are AATAGC and ACTCCT with average copy numbers of 130 in IA and 87 in IC, respectively (but which are each still present but rare in the other genotype from that region).

We found a range of 104–148 k-mers that appeared at least twice in all SC, EC, and MA lines of a particular genotype and observed 283 unique k-mers across all genotypes. There were 13 highly abundant k-mers with an average copy number ≥100 across the SC lines of all genotypes (table 1) which ranged in length from 1 to 6 bp. In contrast, *D. pulex* has 39 repeats with an average copy number ≥100 (Flynn et al. 2017) and these k-mers ranged in length from 1 to 20 bp. Of the highly abundant *D. magna* k-mers, 12 out of 13 exist in the *D. pulex* data set, whereas only 19 of the 39 highly abundant *D. pulex* k-mers exist in our *D. magna* data set. In both species, the most abundant k-mer was the 1-mer A and followed by the 5-mer AACCT, but the copy number was much higher in *D. pulex* for both (table 1). As noted previously, AACCT is likely an ancestral telomeric repeat in Arthropods that is present in several crustaceans (*Daphnia pulicaria*, *Gammarus pulex*, and *Penaeus semisulcatus*) and insects (Okazaki et al. 1993; Sahara et al. 1999; Schumpert et al. 2015). On average, 30% of the total k-mer base pairs were composed of AACCT in *D. magna* and 26% in *D. pulex* (Flynn et al. 2017). K-mers C, AAC, and AAG had similar copy numbers between the two species, whereas the remaining eight high abundance k-mers in *D. magna* had lower copy numbers or were absent in *D. pulex* (table 1).

**Table 1.** Normalized Copy Number of Highly Abundant k-mers for Each SC Line from Six Genotypes of *Daphnia magna* Collected from Three Locations, Finland (F), Germany (G), and Israel (I).

k-mer	*D. magna*						*Daphnia pulex*
	FA	FC	GA	GC	IA	IC	
A	21,847	22,096	19,529	22,181	12,965	18,481	85,440
AACCT	9,767	8,083	4,399	6,347	6,799	11,390	48,905
AAG	7,325	6,333	5,047	8,817	5,178	7,292	1,893
C	7,246	4,892	2,661	5,796	2,495	2,870	4,982
AG	3,900	2,528	4,231	3,001	3,179	4,687	376
ACTAT	2,136	2,462	1,569	1,678	1,316	1,956	—
AAAAC	988	1,376	627	1,236	1,296	676	8
AC	622	567	604	624	948	971	269
AAC	285	278	324	360	354	440	279
AGC	177	168	209	180	167	150	55
AAT	190	150	188	169	134	142	4
AT	151	159	113	165	149	134	2
AACAGG	53	105	171	110	128	232	31

a Copy number for *D. pulex* represents the mean across their six non-MA lines.

For the k-mers with at least two copies across all lines of a genotype, the distribution of repeat unit lengths was similar across the six genotypes (table 2). K-mers with short lengths tend to have higher copy number than longer k-mers. We observed an abundance of k-mers with lengths divisible by three (i.e., 3-, 6-, 9-, 12-, 15-, and 18-mers) and an abundance of 5-mers. K-mers with lengths 5, 6, 12, and 15 were also very common in *D. pulex*. However, *D. pulex* contained an abundance of 10- and 20-mers (15 and 50, respectively), which we did not observe in *D. magna*.

**Table 2.** Count and Average Copy Number for k-mers of Different Lengths (*k*) Found in Each Genotype of *Daphnia magna* Assayed in this Experiment (Also with *Daphnia pulex* Data from Flynn et al. [2017]).

*D. magna*								*D. pulex*	
# k-mers Across Lines for each genotype									
*k*	FA	FC	GA	GC	IA	IC	Mean copy number^a^	# k-mers across Lines	Mean copy number^a^
1	2	2	2	2	2	2	13,578	2	42,954
2	3	3	3	3	3	3	1,361	2	326
3	9	9	8	9	9	9	668	5	2,159
4	8	6	3	3	5	6	31	4	267
5	11	13	9	10	12	10	938	10	8,953
6	14	15	8	18	17	19	31	10	148
7	3	1	1	3	5	5	45	1	189
8	2	2	2	3	1	0	7	3	202
9	3	3	6	9	9	8	10	4	11
10	5	4	2	3	5	5	9	15	875
11	6	3	3	2	2	3	21	1	10
12	25	21	17	22	24	19	6	19	128
13	4	4	2	3	8	7	12	5	156
14	2	2	1	1	3	2	6	3	16
15	26	24	16	16	19	21	6	13	15
16	1	1	1	1	0	0	6	1	7
17	1	1	2	2	0	0	4	6	175
18	16	18	16	16	18	16	6	6	17
19	5	5	1	1	2	2	5	2	16
20	2	2	1	1	2	2	5	50	439

a Mean copy number was averaged across all lines for all k-mers of repeat unit length, *k*.

### Microsatellite Mutation Rate Profiles of *D. magna*

Since *k-Seek* outputs the total count of a given k-mer across all locations in the genome, our mutation rates are an estimate of the change in the total copy number of a k-mer, which could have occurred at one or more locations in the genome (see Material and Methods section for details). For each of the six genotypes, we estimated mutation rates for k-mers with at least six copies in the SC line and at least two copies in each of the EC and MA lines. The number of k-mers with estimated mutation rates ranged from 60 to 79 across the six genotypes, which totaled to 144 unique k-mers. Across all six genotypes, 31 k-mers were present in all populations, whereas 20, 22, and 29 k-mers were unique to genotypes of Finland, Germany, and Israel, respectively. We observed that the absolute value of the mutation rate, |U_*i*__,__*j*_| was strongly positively correlated with the initial copy number of the k-mer in the SC line for each genotype (average correlation = 0.75, supplementary fig. S1, Supplementary Material online). This was expected because k-mers with higher representation in the genome likely represents a larger mutational target. To remove this correlation, we divided the mutation rate of each k-mer by its initial abundance to obtain an estimate of per copy mutation rate, u_*i*__,__*j*_ (average correlation = 0.0031, supplementary fig. S1, Supplementary Material online). It is important to note, the program we used (*k-Seek*) estimates the copy number across all arrays of a particular k-mer and thus our mutation rates are an estimate of the net change in copy number due to increases and decreases at all arrays, rather than an estimate of array length changes (see Materials and Methods section for details). Thus, a positive (negative) value for the genome-wide or per copy mutation rate does not mean that the particular k-mer only experienced increases or expansions (decreases or contractions) in copy number, rather it means that the net effect of mutation was to increase (decrease) copy number.

**Fig. 2.**
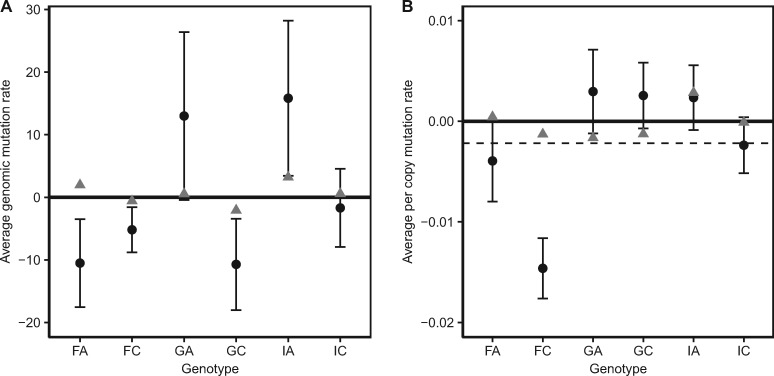
Mean (±SE) genomic mutation rate (*A*) and per copy mutation rate (*B*) for each genotype from six genotypes of *Daphnia magna* collected from three locations, Finland (F), Germany (G), and Israel (I). Black circles and gray triangles represent MA and EC lines, respectively. The dashed line represents the mutation rate of MA lines averaged across all genotypes.

We observed high levels of variation in microsatellite mutation rates for *D. magna*, ranging from negative (net decrease in copy number for a given k-mer) to positive (net increase in copy number for a given k-mer), even among lines from the same genotypes. Across all MA lines and k-mers, the genome-wide mutation rate (U_*i*__,__*j*_) ranged between −1,103 and 2,370 copies per generation and the per copy mutation rate (u_*i*__,__*j*_) ranged between −0.19 and 0.33 copies per initial copy number per generation. We found that mutation rates varied considerably across the six genotypes, but not consistently between genotypes of the same population. Figure 2 shows the k-mer mutation rates (both U_*j*_, u_*j*_) averaged across all k-mers for each genotype. For both U_*j*_ and u_*j*_, FA, FC, and IC had negative mutation rates (meaning a decrease in copy number), whereas GA and IA had positive mutation rates, on average. GC had negative U_*j*_, but positive u_*j*_, on average, which indicates that there was one or more k-mers that possessed low negative per copy mutation rates (u_*j*_), but were abundant enough to cause the average of U_*j*_ to be negative (which weights u_*j*_ by the abundance of k-mer *j*).

We used the absolute value of the per copy mutation rates (|u_*i*__,__*j*_|) to examine the magnitude of k-mer copy number change. Across all MA lines and k-mers, the average absolute per copy mutation rate ranged from 0.0000042 to 0.33 and had a mean of 0.029. EC lines had a lower rate of k-mer copy number change with an average 0.0049, suggesting that selection indeed constrained the rate of k-mer copy number change in these large population controls.

Overall, k-mer mutation rates were higher in magnitude and more variable in *D. magna* than in *D. pulex*. To compare to the *D. pulex* data set, we applied a similar filter (requiring that each k-mer considered have at least two copies in all MA lines and at least six copies across all non-MA lines [as a proxy for the ancestor]). For the 121 k-mers for which we were able to estimate absolute per copy mutation rates, |u_*i*__,__*j*_|, the mean was 0.004 copies per copy per generation (ranging from 0 to 0.053), which is an order of magnitude lower than the average rate for *D. magna* MA lines. Only 21 of the 121 *D. pulex* k-mers were present in *D. magna* (supplementary fig. S3, Supplementary Material online), and the average |u_*i*__,__*j*_| for these 21 k-mers in *D. pulex* and *D. magna* was 0.0041 and 0.031 copies per copy per generation, respectively. Furthermore, the coefficients of variation in |u_*i*__,__*j*_| for these 21 k-mers were consistently lower in *D. pulex* than in the *D. magn*a genotypes (supplementary fig. S3, Supplementary Material online).

### Mutation Rate Variation Based on Features of the k-mer

**Fig. 3.**
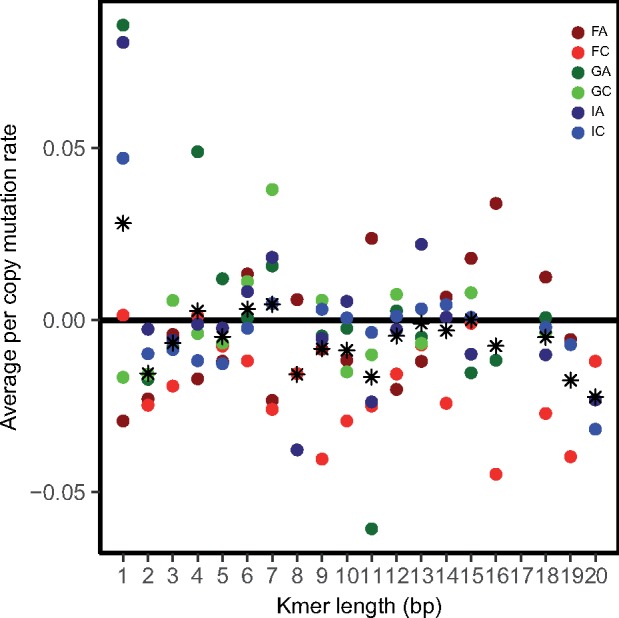
Means of k-mer per copy mutation rate for each genotype and length of k-mer from six genotypes of *Daphnia magna* collected from three locations, Finland (F), Germany (G), and Israel (I). The asterisk symbol represents the average value across the six genotypes.

Per copy mutation rates of individual k-mers, u_*j*_, varied greatly between k-mers of different lengths (fig. 3, supplementary fig. S4, Supplementary Material online). Figure 3 shows the average value of u_*j*_ across k-mers of the same length (*k*) for each genotype, which we define as u_*j*_(*k*). This value [u_*j*_(*k*)] can be positive or negative at most k-mer lengths, depending on the particular genotype. Per copy mutation rates were most positive in 1-mers and tend to be more negative in k-mers with k ≥ 8. However, fitting a linear model (lm (u_*j*_ ∼ *k*)), for each genotype did not show that per copy mutation rate was significantly correlated with k-mer length (supplementary table S1 and fig. S4, Supplementary Material online), likely because of the considerable variation in mutation rates even among k-mers of the same length. Indeed, Kruskal–Wallis tests across k-mers of the same length show significant variation in u_*j*_ within genotypes for most k-mer lengths (supplementary table S2 and fig. S4, Supplementary Material online), in addition to the variation in mutation rates at each k-mer length observed between genotypes illustrated in figure 3.

**Fig. 4.**
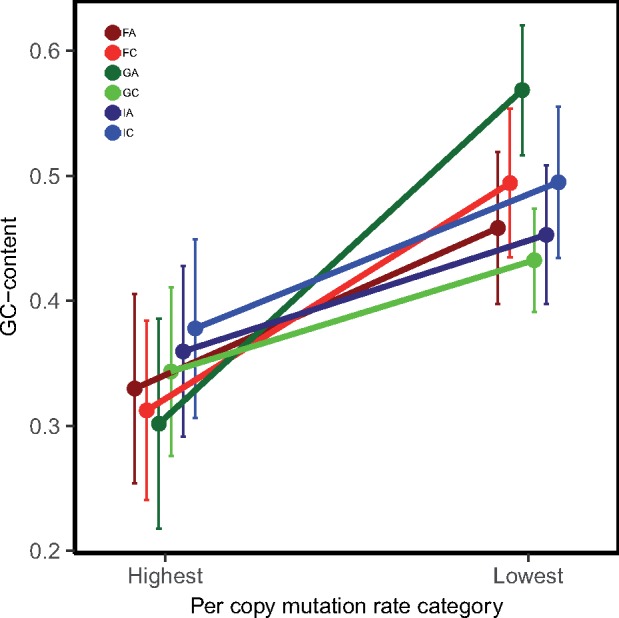
GC-content (mean ± SE) of k-mers with the top highest and lowest absolute per copy mutation rates, |u_*j*_|, from six genotypes of *Daphnia magna* collected from three locations, Finland (F), Germany (G), and Israel (I).

We also examined whether GC-content may have affected mutation rates. K-mers containing higher levels of GC may have a lower propensity to undergo mutation because GC pairs forms a more stable bond than AT pairs. To measure the propensity for mutation, we calculated the absolute values of per copy mutation rates (|u_*j*_|), which combines the rates of k-mer copy increases and decreases, for k-mers with repeat unit lengths longer than 3 bp. For each genotype, we examined the GC-content of the ten k-mers with the highest and the ten with the lowest absolute per copy rates (|u_*j*_|; fig. 4, supplementary table S5, Supplementary Material online). We excluded k-mers less than 3 bp long because these k-mers will have extreme values of GC-content; including the 1-mers and 2-mers did not qualitatively change our results. We observed that average |u_*j*_| for the k-mers with the highest rates were at least four times higher than k-mers with the lowest rates (supplementary table S4, Supplementary Material online). As predicted, the k-mers with lower |u_*j*_| tend to possess higher GC-content across all genotypes. A two-way ANOVA for GC-content with genotype and mutation rate category (high vs. low) as factors revealed that GC-content significantly differed between k-mers with the highest and lowest mutation rates (supplementary table S6, Supplementary Material online).

### Linking Variation in Microsatellite Landscapes and Microsatellite Mutation Rates

**Fig. 5.**
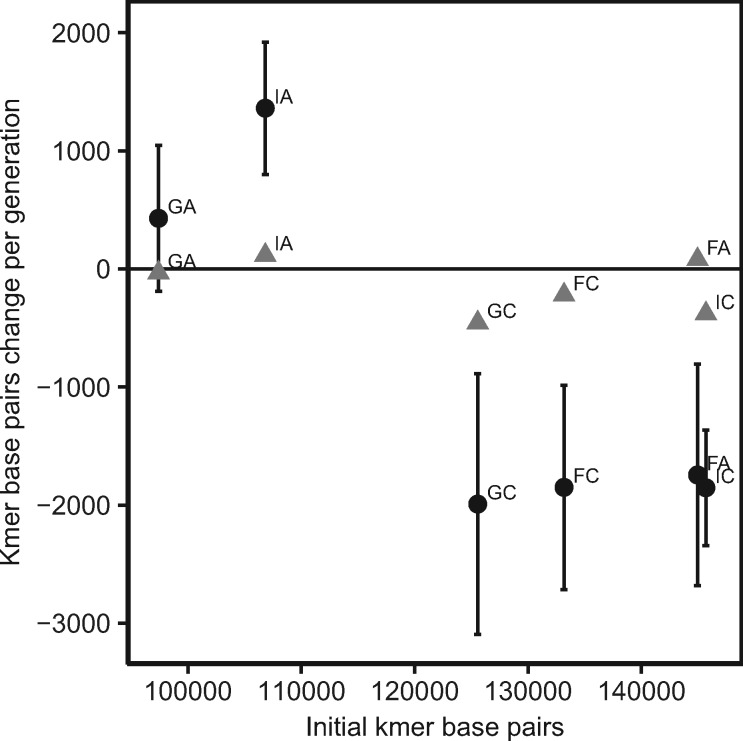
Mean (±SE) k-mer base pair change per generation for six genotypes of *Daphnia magna* collected from three locations, Finland (F), Germany (G), and Israel (I). Black circles and gray triangles represent MA and EC lines, respectively.

The total amount of change in k-mer content during MA is not consistent within or between populations (fig. 5). Both genotypes from Finland (FA, FC) experienced a reduction in k-mer content per generation, on average, whereas one genotype from Germany and Israel (GA and IA) experienced an increase in k-mer content while the other experienced a decrease (GC and IC). Since the MA lines of GA and IA experienced the greatest increases in k-mer base pairs, on average, we expected these two genotypes would contain the highest amount of k-mer content, overall, but the opposite is true (fig. 5). The SC lines of GA and IA contained the lowest k-mer content initially, but exhibit the highest rates of increase due to mutation. In contrast, the SC lines of FA, FC, GC, and IA contained the highest k-mer content and showed the greatest declines in k-mer content during MA. We tested if the change in GC-content of the microsatellite portion of the genome also varied based on starting GC-content level, but observed no significant relationship (supplementary fig. S5, Supplementary Material online). Thus, instead of exhibiting strong differences based on population-of-origin (supplementary fig. S7, Supplementary Material online) or features of individual k-mers, the major differences in mutation rate profiles appear to depend on features of the genome-wide k-mer content, overall.

**Fig. 6.**
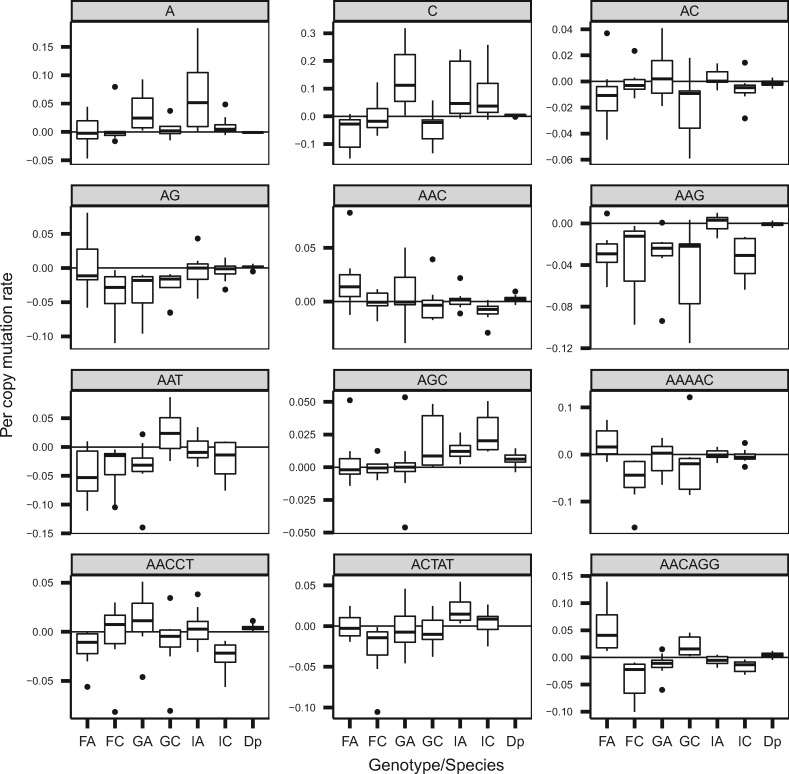
Per copy mutation rate for the 12 k-mers with the highest copy number across for six genotypes of *Daphnia magna* collected from three locations, Finland (F), Germany (G), and Israel (I). Dp represents the per copy mutation rate for *Daphnia pulex*. Points indicate lines with per copy mutation rates less than Q1 − 1.5*IQR or larger than Q3 + 1.5*IQR. Q1, Q3, and IQR represent the first quartile, third quartile, and the interquartile range, respectively.

As alluded to previously, average k-mer mutation rates ranged from positive to negative and varied between genotypes without being consistent within populations (fig. 2). We can examine this in more detail by focusing on the 31 k-mers with mutation rate estimates across all six genotypes. Kruskal–Wallis tests show significant variation in u_*j*_ across genotypes for all but two of the k-mers (fig. 6, supplementary table S3, Supplementary Material online). If k-mer mutation profiles were similar within populations, we would expect high correlations in u_*j*_ for genotypes from the same population, however this was not observed (supplementary table S6, Supplementary Material online). IA and IC possessed a relatively strong positive correlation (0.67) in u_*j*_, but they also shared a strong correlation with GA (IA–GA: 0.60, IC–GA: 0.63). Furthermore, this correlation was mainly driven by their shared high positive u_*j*_ for the k-mer C (fig. 6). Removing the k-mer C reduced the pairwise correlations (IA–IC: 0.33, IA–GA: 0.47, IC–GA: 0.40). We also performed a PCA using the u_*j*_ of the 31 shared k-mers and did not find evidence of clustering by population-of-origin based on principle components one and two (supplementary fig. S6, Supplementary Material online). Including all 144 k-mers (i.e., genotype-specific k-mers) in the PCA improved clustering slightly (supplementary fig. S7, Supplementary Material online).

## Discussion

Repetitive regions of the genome, once overlooked, are now known to be a large and dynamic component of the genome, often responsible for large proportions of the genetic variation among individuals and species. Microsatellite loci, in particular, are known to exhibit elevated mutation rates compared with unique sequences, and have been shown to be important components of the genome in a variety of functional contexts ranging from disease risk to speciation (Shah et al. 2010; Gemayel et al. 2012; Hannan 2018). The goal of this study was to quantify intraspecific and interspecific variation in the microsatellite landscape and microsatellite mutational dynamics in *Daphnia* at microsatellite loci composed of k-mers ranging from 1 to 20 bp appearing as tandem repeats spanning at least 50 bp. As mentioned in the Results section and described in the Supplementary Material online, an additional analysis using a lower threshold of 10 bp minimum span did not significantly affect our estimates of mutation rates (supplementary fig. S9, Supplementary Material online). Based on our analysis of six *D. magna* starting control lines (SC), 47 MA lines and 12 non-MA extant control lines (EC), we were able to characterize the k-mer profile of our *D. magna* lines based on 283 k-mers, and were able to estimate mutation rates for 144 k-mers. Using an MA experiment and genome-wide approach, our estimates of k-mer mutation rates provide a lower-bound estimate of the net change in k-mer copy number due to mutations of all types across all microsatellite loci.

### Microsatellite Landscapes Are Distinct among Genotypes, Populations, and Congeners

Our results clearly show distinctive microsatellite landscapes among the six genotypes from the three different populations of *D. magna* (fig. 1*A* and *B*). Using the abundance of 100 k-mers present in all lines, we observed clusters of genotypes by their population-of-origin (fig. 1*A*). In addition, within each population, we observed that MA and EC lines form distinct clusters based on genotype (fig. 2). These results are conservative, and including k-mers unique to genotypes would only strengthen the clustering. For the k-mers with presence/absence polymorphism across genotypes, the vast majority were low in copy number suggesting that they arose relatively recently. In contrast, microsatellite analysis for six genotypes of *C. reinhardtii* found many k-mers with hundreds of copies in some genotypes but absent or rare in others (Flynn et al. 2018). Our analyses reveal that the k-mer content of *D. magna* exhibits high levels of intraspecific variation, even within populations.

The microsatellite profile of *D. magna* is distinct from that of the only previously examined congener, *D. pulex* (Flynn et al. 2017), which has a much higher proportion of microsatellite content in its genome (tables 1 and 2). In *D. pulex*, the most abundant k-mers occurring in the genome tend to be shorter repeat units, with the exception of some longer repeats, such as the known arthropod telomeric sequence (AACCT)_n_ (Okazaki et al. 1993). However, there are many k-mers that are unique to each species and, for k-mers that are shared, many differ greatly in copy number (tables 1 and 2). *Daphnia magna* is enriched for k-mers with unit lengths that are multiples of three (table 2). It is possible that these k-mer lengths are more tolerated by selection because they are less likely to cause frameshift mutations within coding regions (Metzgar et al. 2000). *Daphnia pulex*, on the other hand, is enriched for k-mers with unit lengths that are multiples of five, as has also been reported in *Dr. melanogaster* (Wei et al. 2014). The distinctive microsatellite landscapes observed both within and between these species invite the question—do microsatellite mutation dynamics vary widely and thus explain the accumulated differences observed among genotypes, populations, and species over long time periods?

### Microsatellite Mutation Rates Vary among Genotypes and between Species

Mutation rates (both genome-wide increases and decreases in copy number across k-mers [U_*ij*_] and per copy adjusted rates [u_*ij*_]) vary widely among genotypes (fig. 2). For per copy adjusted rates, the two genotypes collected from Finland exhibit declines in average copy number with MA, whereas those from Germany exhibit increases, and the two genotypes collected from Israel split, with one genotype having an overall positive per copy mutation rate and one having a negative rate. This level of intraspecific variation in rates has not been reported previously, although this could be an artifact of most studies being conducted on only one or a few genotypes based on the assumption that mutation rate estimates can be generalized. One of the major take-home messages of this study is that intraspecific variation in microsatellite mutation rates is substantial, with some genotypes experiencing increases in k-mer copy number and others exhibiting a decrease in k-mer copies, overall. Across all k-mers and genotypes, on average, copy number change was more often negative than positive (fig. 3) in *D. magna*, which is the opposite of the pattern observed in *D. pulex* (supplementary fig. S8, Supplementary Material online). Such large differences in mutation rate at both the intraspecific level and between congeners may pose a challenge for recent theory aimed at explaining the evolution of the mutation rate across lineages. Specifically, the “drift barrier hypothesis,” which posits mutation rates can only be driven down by selection to the extent possible based on the effective population size, at which point they cannot be lowered further due to the relative power of genetic drift which permits mutations that increase (or maintain) the rate to persist by chance (Lynch 2010). Without major differences in life history, physiology, body size, or effective population size, one would not predict an order of magnitude difference in mutation rate between *D. magna* and *D. pulex* if a drift barrier exists. Estimates of effective population size (*N*_e_) for these two species (based on calculations using an identical mutation rate, however) point to only a 3-fold difference in *N*_e_ (Haag et al. 2009), suggesting further investigation of intra- and inter-specific variation in mutation rates will be essential for testing the explanatory potential of the drift barrier hypothesis to explain mutation rate variation within and between species.

### Microsatellite Mutation Rates as a Function of k-mer Length and k-mer Content

We examined the variation in k-mer abundance based on features of the k-mers themselves—both length and GC-content. There was no relationship between length and copy number change (fig. 3), with one major exception—1-mers exhibit the highest positive mutation rate, on average. We observed that k-mers with high GC-content tend to have lower mutation rates (fig. 4 and supplementary table S4, Supplementary Material online). This is not a surprise in that the three hydrogen bonds holding GC pairs together might be less prone to mutation than regions that are AT-rich, given there are only two hydrogen bonds between As and Ts (Calabrese and Durrett 2003; Fan and Chu 2007).

### The Relationship between Microsatellite Landscape and Mutation Rates

We explored the relationship between initial k-mer content in the genome and the mutation profiles for each genotype to determine if the microsatellite landscape could be explained by the patterns of MA observed in the laboratory. Given that we observed a strong positive correlation between microsatellite abundance and absolute mutation rates (leading to the calculation of per copy mutation rates for our subsequent analyses), we were surprised to find that genotypes with high initial genome-wide k-mer content exhibit greater decreases in microsatellite content (total change in bp) as a result of mutation than genotypes with low initial k-mer content, which exhibit greater increases in the bp contributed by microsatellites during MA (fig. 5). The context-dependency of microsatellite mutation dynamics has been reported previously, for example studies have shown that longer arrays tend to decrease in length whereas shorter arrays tend to increase (Xu et al. 2000; Lai and Sun 2003). However, the dependency of a mutation bias toward increasing or decreasing microsatellite content on the initial total amount of microsatellites DNA has not yet been reported to our knowledge.

If starting microsatellite content does, indeed, determine the direction of copy number change as observed here *within* a species, we would predict that the extremely high microsatellite content (10-fold higher than in *D. magna*) reported for *D. pulex* in Flynn et al. (2017) would correspond with declines in copy number during MA. This is, in fact, the opposite of what was reported—*D. pulex*, overall, shows a bias toward copy number increases (Flynn et al. 2017), whereas *D. magna* shows an overall bias toward decreasing copy number (illustrated by the asterisks in fig. 3; supplementary fig. S8, Supplementary Material online). This observation, combined with the observation that *D. magna* exhibit a 10-fold higher overall rate of microsatellite mutation, presents a genomic puzzle. It is possible that the copy number increase bias, combined with lower mutation rates, has led to a slow but tolerable accumulation of higher k-mer content in the *D. pulex* genome over time. A similar explanation has been posited for plant versus animal mitochondrial genomes, where low mutation rates and a mutation bias toward insertions may have led to the tolerable accumulation of noncoding DNA resulting in, typically, much larger organellar genomes than in animals (Lynch et al. 2006).

### Comparison between *D. pulex* and *D. magna*

As mentioned, there were a few differences that limit our ability to make a direct comparison between our results for *D. magna* and those in Flynn et al. (2017) for *D. pulex* without some caveats (i.e., coverage differences and read length differences). Since *k-Seek* only counts tandem repeats spanning at least 50 bp on a read, the shorter reads and lower coverage in Flynn et al. (2017) may make it more difficult to detect k-mer copies, especially of longer k-mers, in their study. However, k-mer content reported in *D. pulex* was actually much higher than in *D. magna* and there was no obvious bias toward detecting shorter k-mers (table 2). In fact, Flynn et al. (2017) detected many more 10- and 20-mers in *D. pulex* than we found in *D. magna*. An additional difference was that Flynn et al. (2017) used the average copy number of k-mers in non-MA lines as a proxy for k-mer estimates for the ancestral line as a baseline for calculating rates. In our data set, non-MA lines experienced k-mer content change at a much slower rate than MA lines, suggesting that although non-MA lines serve as a relatively good proxy for ancestral lines (fig. 5, supplementary fig. S8, Supplementary Material online), this could contribute to a slight underestimate of rates. Although we would not expect this to explain the order of magnitude difference in mutation rates between *D. pulex* and *D. magna* (which we observed even when only comparing their 21 shared k-mers) and the differences between species in overall k-mer content, the differences in the studies likely affect the sensitivity of each analysis.

### Conclusions

We observe major differences in the microsatellite landscapes accumulated over long time periods between genotypes and populations of *D. magna*, and between this species and the previously studied congener, *D. pulex*. High levels of differentiation in repeat landscapes were also previously reported, among both populations of *Dr. melanogaster* (Wei et al. 2014) and among species of *Caenorhabditis* worms (Subirana et al. 2015). Given microsatellite landscapes are shaped not only by mutational inputs, but also by selection and drift, this is not a major surprise. Our results beg the question whether mutation rate differences or differential impacts of other evolutionary forces play a greater role in explaining these observed differences.

Although we observe high levels of variation in the mutation rates among genotypes and k-mers in *D. magna*—with some exhibiting net increases in copy number and others exhibiting net decreases in copy number—the variation does not mirror the differences seen in landscapes over long time periods. In fact, genotypes with the lowest k-mer content had the highest rates of copy number increase, and vice versa. Thus, it is clear the differences in microsatellite landscapes within *D. magna* are not being driven purely by mutational inputs, but instead likely reflect the interplay of mutation, selection, and drift, potentially resulting in an equilibrium with respect to individual loci (Kruglyak et al. 1998) or overall repeat content in the genome (Petrov 2002). Overall, genotype- and k-mer-specific variation in mutation rates (fig. 6) reveals a large range in terms of mutation rates in this species, and suggests that there is abundant variation upon which natural selection could act to shape mutation rates within *D. magna*.

In addition to investigating intraspecific variation and the degree to which long-term patterns of MA would correspond to short-term mutation rates, another goal of this study was to assess the degree to which mutation rates are consistent between closely-related species. Overall, we observe much higher (10-fold) absolute microsatellite mutation rates in *D. magna* (regardless of bias toward increasing or decreasing k-mer copies), than those reported for *D. pulex* (Flynn et al. 2017). Importantly, we see a mutation bias toward decreasing copy number in *D. magna* (reflected by the lower overall k-mer content in this species), relative to the increase bias reported for *D. pulex*, which corresponds to the much higher level of k-mer content reported for that species (Flynn et al. 2017). Although it is possible that a higher effective population size (*N*_e_) of *D. pulex* (Haag et al. 2009) allows selection to more efficiently lower the mutation rate in this species, it seems unlikely that this difference could explain the order of magnitude difference in mutation rate observed. Alternatively, the bias toward a decrease in copy number observed in *D. magna* may make the high mutation rates more tolerable, even under a similar selective regime, assuming increasing k-mer content in the genome is deleterious.

It will be interesting to investigate other aspects of the mutational profile (e.g., base substitution and indel rates) of these two species in order to determine what other major differences in mutation dynamics are observed. Future studies examining the rates of contraction and expansion across k-mers, as well as the differential mutability of microsatellites within, near, or far from protein-coding regions will also yield insights into the intragenomic variability in mutation rates. Although it has been common heretofore to generalize empirical estimates of mutation rates from experiments using a single genotype from a model species, the level of intra- and inter-specific variation reported here suggests caution should be taken when doing so. Once we have a more complete picture of the rates, directionality, and consequences of mutation within and among species and across the genome, we will be able to better predict adaptive potential, frequencies of genetic disease, and rates of evolution for individuals, populations, and across taxa.

## Materials and Methods

### Study System

The six *D. magna* genotypes used to initiate this experiment were collected along a latitudinal gradient that captures a range of environmental variation including temperature and photoperiod. Specifically, two unique genotypes from each of three populations (Finland, Germany, and Israel) were used for the MA experiment (see supplementary fig. S10, Supplementary Material online, for schematic). The stock cultures for each genotype were maintained in 250 ml beakers containing 175–200 ml of Aachener Daphnien Medium (ADaM; Klüttgen et al. 1994) under a constant photoperiod (16L:8D) and temperature (18 °C), and fed the unicellular green alga *Scenedesmus obliquus* ad libitum (2–3 times per week).

### Mutation Accumulation Experiment

MA lines (*n* = 47) were initiated from clonally-produced offspring of a single female isolated from the stock cultures of each of the six genotypes described above. The MA protocol used here has been described previously (Eberle et al. 2018). Briefly, MA lines were initiated by placing a single clonally-produced female in a 250 ml beaker containing 100 ml of ADaM supplemented with *S. obliquus* at a concentration of 600,000 cells/ml. All MA lines were maintained in environmental conditions identical to the control lines (16L:8D, 18 °C). The food/media mixture in each beaker was replaced once per week, and each line was fed a prescribed volume of concentrated *S. obliquus* 3 days after the media replacement to reset the algal cell concentration in the beaker to 600,000 cells/ml. From generation to generation, each MA line was propagated via single-progeny descent by taking a single juvenile offspring from the second clutch of the previous generation. A series of backups were maintained in parallel with the focal lineages in the event that the single individual intended to be used to establish the next generation died before reproduction or was a male. Tissue samples from each of the MA lines were isolated every five generations and, at the end of the MA period, the samples taken from lines with the greatest number of generations of MA were used for DNA extraction and sequencing (2–26 generations, with an average of 12.4 generations per line).

Two types of controls were used in this study. First, from immediate descendants of the individuals used to establish the MA lines (at the beginning of the MA period), we collected 5–20 animals and froze tissue (referred to hereafter as “starting controls” [SCs]). Second, we used immediate descendants to establish populations that were then maintained at a large size (referred to hereafter as “extant controls” [ECs]) for the duration of the experiment (approximately 2 years). In the large population controls, mutations may occur, but are more likely to be purged by selection due to competition among clones (relative to the MA lines, where clones are reared individually and experience no competition). The paucity of mutations observed in EC lines (see fig. 2) is consistent with the idea that the MA protocol (e.g., bottlenecking lineages each generation by transferring a single individual) does, indeed, minimize selection and allow for the accumulation of mutations. The two EC lines were maintained in separate 3 l jars containing 2 l of ADaM, under constant temperature (18 °C) and photoperiod (16L:8D), and fed the unicellular green alga *S. obliquus* ad libitum. The media in the jars was replaced every 2–3 weeks, and individuals in the two replicate jars were mixed to maintain as much genetic homogeneity among the jars as possible. Population sizes in the ECs varied between several hundred to a few thousand individuals. For all lineages sampled, individuals collected for DNA extraction were placed in a 1.5 ml microcentrifuge tube, frozen in liquid nitrogen, and stored at −80 °C until DNA extraction.

### DNA Extraction and Sequencing

Five clonal individuals from each MA line and controls (1 SC and 2 EC per genotype) were flash frozen for DNA extractions. DNA was extracted (two extractions per line with five animals each) using the Zymo Quick-DNA Universal Solid Tissue Prep Kit (No. D4069) following the manufacturer’s protocol (DNA from a few samples was also extracted with the Qiagen DNeasy Blood and Tissue Kit, No. 69504). DNA quality was assessed by electrophoresis on 3% agarose gels and DNA concentration was determined by dsDNA HS Qubit Assay (Molecular Probes by Life Technologies, No. Q32851). The Center for Genome Research and Biocomputing at Oregon State University generated 94 Wafergen DNA 150 bp paired-end libraries using the Biosystems Apollo 324 NGS library preparation system. Quality was assessed using a Bioanalyzer 2100 (Agilent Technologies, No. G2939BA) and libraries were pooled based on qPCR concentrations across 16 lanes (2 runs). Libraries were sequenced on an Illumina Hiseq 3000 (150 bp PE reads) with an average insert size of ∼380 bp to generate approximately 50× coverage genome-wide for each sample (supplementary table S7, Supplementary Material online). *All sequence data generated will be submitted to GenBank upon acceptance of the article.*

### Tandem Repeat Quantification

Sequenced reads from all lines were trimmed of adapters and decontaminated to remove mitochondrial sequences. Overlapping reads were merged with BBmerge (Bushnell et al. 2017). To quantify tandem repeats, the reads were input into the program *k-Seek* (Wei et al. 2014). The program *k-Seek* detects tandem repeats (k-mers) of 1–20 bp, requiring that the k-mers repeat tandemly over at least 50 bp on a given read, allowing for 1 bp mismatch per repeat unit. Offsets and reverse complements of each k-mer are combined and the output is the total count across all reads for each k-mer. Because *k-Seek* has the requirement that tandem repeats span at least 50 bp, the threshold number of repeat units required for detection decreases as the length of the repeat unit increases (e.g., 1-mers require at least 50 repeats to be detected whereas 5-mers only require a minimum of 10 repeats to be detected). On the other hand, the maximum number of repeat units on a single read decreases as the k-mer length increases. We do not observe a detection bias toward k-mers with longer or shorter lengths (table 2), which suggest that k-mer length is not the main determinant of k-mer detection. Another consequence of the requirement that tandem repeats must span at least 50 bp is that we do not detect a large number of shorter tandem arrays, which is especially problematic for smaller k-mers. Our analysis using *k-Seek* with a modified threshold (10 bp) for minimum spanning distance revealed an average of 41% increase in the k-mer base pairs detected (for k-mers with *k* < 10), however, the mutation rates for these k-mers were largely unaffected. To make sure our results were comparable to other studies (e.g., Wei et al. 2014; Flynn et al. 2017, 2018), we performed our analysis using the default *k-Seek* threshold of 50 bp minimum span, but it is important to note that the microsatellite content reported is a subsample of the genome which meets this criterion and rates calculated are based on this subsampling.

To compare across samples, we normalized k-mer counts by dividing copy number counts by the median sequence depth matched by the GC-content of the k-mer. We first constructed de novo reference genomes for each of the six *D. magna* starting genotypes using Spades (Bankevich et al. 2012). Reads from each line were mapped to their corresponding reference genome using BWA default settings (Li and Durbin 2009). Following Flynn et al. (2017), output BAM files were input into a custom script to calculate the coverage depth at each uniquely mappable base and the GC-content of the nearby region (https://github.com/jmf422/Daphnia-MA-lines; Last accessed on November 12 2018). We then group each base pair based on their nearby GC-content in the following bins: {0–0.3, 0.3–0.35, 0.35–0.4, 0.4–0.45, 0.45–0.5, 0.5–0.55, 0.55–0.6, 0.6–1}; we used wider bins for GC-content ≤0.3 and >0.6 because there were many fewer sites containing very low and high GC-content. For each GC-content bin, we then determined the median base pair depth for use as the normalization factor. For each line, we normalize the total count of each k-mer by dividing the total count by the normalization factor that corresponds with the GC-content of that k-mer. This normalization approximates the copy number per 1× coverage of each k-mer, which for simplicity we will refer to as the “copy number.”

Note that, after normalization, the total base pairs covered by a particular k-mer in a particular line (i.e., k-mer length * normalized copy number) can fall below 50 bp even though *k-Seek* required tandem repeats span at least 50 bp. This is due to an overcorrection by our normalization method (e.g., for a 1-mer that spans exactly 50 bp in the genome, there may be many reads that encompass the whole 50 bp array, but also many reads that only encompass a portion of the array). In this case, *k-Seek* will not count the number of 1-mer repeats in reads that do not contain the full 50 bp array, because it falls below its threshold array length requirement. However, those reads are still counted toward our normalization factor. Due to this, the normalized copy number can fall below 50 for these 1-mers. This overcorrection due to normalizing total copy number by the average (or median) coverage is present in all previous analyses that utilized *k-Seek* (Wei et al. 2014; Flynn et al. 2017, 2018). Overall, this would cause an underestimation of the total k-mer content in the genome.

### Mutation Rate Estimation

*k-Seek* outputs the total count of a given k-mer across all locations in the genome as long as the requirements mentioned above are met. Thus, for our estimation of mutation rates, we define mutation as the change in the total copy number of a k-mer, which could have occurred at one or more locations in the genome. For each genotype, we restrict our mutation rate analysis to k-mers where the SC line had at least six copies and each of the MA lines had at least two copies. This allowed us to estimate mutation rates for 71 k-mers, on average, for each of the six genotypes. We define the *genomic mutation rate* of k-mer *j* in MA line *i* as U_*i*__,__*j*_ = (c_*i*__,__*j*_ − c_SC,__*j*_)/g_*i*_, where c_*i*__,__*j*_ and c_SC,__*j*_ represent the copy number of k-mer *j* at MA line *i* and the SC line, respectively, and g_*i*_ represent the number of MA generations for MA line *i*. We found that the absolute value of the genomic mutation rate was strongly correlated with the abundance of the k-mer in the SC lines (supplementary fig. S1, Supplementary Material online). This was not surprising because highly abundant k-mers likely represent a larger mutational target. To account for differences in the initial abundance of k-mers, we define the *per copy mutation rate* of k-mer *j* in MA line *i* as u_*i*,__*j*_ = U_*i*,__*j*_/c_SC__,__*j*_. We define the overall genomic and per copy mutation for k-mer *j* of a genotype as U_*j*_ and u_*j*_, respectively, which is calculated by taking the average U_*i*,__*j*_ and u_*i*,__*j*_ across all MA lines of the genotype. We calculated mutation rates for EC lines in the same way as we did for MA lines. To estimate the number of generations that each of the EC lines were maintained, we divided the length of the experiment (830 days) by their estimated generation time. In the Supplementary Material online, we also describe our analysis with the modified version of *k-Seek* that only requires microsatellite loci span at least 10 bp (instead of 50 bp) which shows that including these shorter loci does not significantly affect estimates of mutation rates (supplementary fig. S9, Supplementary Material online).

### Comparison to *D. pulex*

Throughout our study, we compare our *D. magna* microsatellite results to previously published results based on a data set from *D. pulex* MA lines (Flynn et al. 2017). Briefly, Flynn et al. (2017) examined the microsatellite content from 28 MA lines and 6 non-MA lines that were all initially generated from a single ancestral genotype. Next generation sequencing was done following an Illumina Nextera library preparation (10× coverage, 100 bp PE reads). They analyzed k-mers copies using *k-Seek* and normalized copy number estimates as we described above (we used their study as a guide for our copy number normalization steps). In addition to shorter read lengths and lower coverage depths of sequencing, another difference in the study is the controls: they did not sequence their initial ancestral genotype, but used the average copy number of the six non-MA lines as a proxy for the ancestral state.

## Supplementary Material

Supplementary data are available at *Molecular Biology and Evolution* online.

## Supplementary Material

msz118_Supplementary_Data
