# Supplementary material for: Intraspecific Variation in Microsatellite Mutation Profiles in Daphnia magna
Source: Mol Biol Evol. 2019 May 11;36(9):1942–54. doi: 10.1093/molbev/msz118 (PMC6934441; doi:10.1093/molbev/msz118)
Supplement: msz118_Supplementary_Data [file msz118_supplementary_data.docx]

**Supplementary Materials**

***Supplementary Methods***

By default, k-Seek requires that tandem repeats span a length of at least 50 bp. This means that motifs (kmers) with short unit lengths (*k*) would need to have more tandem repeats at a locus compared to kmers with longer unit lengths to pass the 50bp threshold (e.g. 1-mers must be repeated at least 50 times, 2-mer must be repeated at least 25 times, 10-mers must be repeated 5 times, etc.). There is some evidence that microsatellite loci containing more repeat units tend to be biased towards contracting in length. If this is true, then for microsatellite loci that span 50bp, the ones composed of kmers with small *k* will tend to contract more often than loci composed of kmers with larger *k* during our MA experiment (because the former must be made up of more repeat units to span 50 bp). Since microsatellite loci that span less than 50 bp will not be detected by k-Seek, we may be overestimating the loss of kmers with short *k*.

To address this potential issue, we modified k-Seek to only require that tandem repeat span a length of at least 10bp. We randomly chose one of our genotypes (IA), performed the same analyses as describe in the manuscript using the modified k-Seek and compared the results to those we found using the default k-Seek; we will call the analysis with the 10bp threshold *t10* and the analysis with the 50bp threshold *t50*. We will only compare results for kmers present in both analysis and has unit lengths < 10 bp because kmers with unit length >= 10 will pass the 10bp threshold without actually being a tandem repeat. To test the hypothesis that kmers with shorter unit lengths, k, will be more strongly affected by the detection bias described above, we took the difference in per copy mutation rates between the t10 and the t50 analysis for each kmer (u_difference_ = u_t10_ – u_t50_) and examined if there was a relationship between u_difference_ and k. The results of this analysis are placed in the foot note of Table S8 and Figure S9.

***Supplementary Tables and Figures***

**Table S1**. Effect of repeat unit length (k) on mutation rate from a linear model, lm(u_j_ ~ k) for six genotypes of *D. magna* collected from three locations, Finland (F), Germany (G) and Israel (I).

| **Genotype** | **D.f.** | **Coefficient** | **P-value** |
| --- | --- | --- | --- |
| FA | 70 | 0.00132 | 0.111 |
| FC | 75 | -0.00066 | 0.232 |
| GA | 58 | -0.00140 | 0.079 |
| GC | 64 | 0.00034 | 0.648 |
| IA | 71 | -0.00103 | 0.154 |
| IC | 77 | 0.00002 | 0.969 |

**Table S2**. P-value from Kruskal-Wallis tests for the effects of kmer repeat unit length on per copy mutation rate for each unit length and genotype for six genotypes of *D. magna* collected from three locations, Finland (F), Germany (G) and Israel (I).

| **k** | **FA** | **FC** | **GA** | **GC** | **IA** | **IC** |
| --- | --- | --- | --- | --- | --- | --- |
| 1 | 0.0639 | 0.3446 | 0.0587 | 0.0460 | 0.6744 | 0.0587 |
| 2 | 0.0256 | 0.0221 | 0.0297 | 0.1831 | 0.1725 | 0.7926 |
| 3 | < 0.0001 | < 0.0001 | 0.0017 | < 0.0001 | 0.0012 | < 0.0001 |
| 4 | 0.0056 | 0.0140 | 0.1791 | 0.0006 | 0.0002 | 0.0290 |
| 5 | 0.0027 | 0.0002 | 0.4714 | 0.0367 | < 0.0001 | 0.0030 |
| 6 | < 0.0001 | < 0.0001 | 0.0032 | < 0.0001 | < 0.0001 | < 0.0001 |
| 7 | 0.0018 | - | - | - | 0.0005 | 0.0005 |
| 9 | - | - | 0.0660 | 0.0159 | 0.0317 | 0.4399 |
| 10 | 0.0023 | 0.0005 | 0.0117 | 0.0147 | < 0.0001 | 0.0023 |
| 11 | 0.0254 | - | - | - | - | 0.0008 |
| 12 | 0.0336 | < 0.0001 | 0.0354 | < 0.0001 | 0.0020 | < 0.0001 |
| 13 | 0.9491 | - | - | - | 0.0823 | 0.0056 |
| 14 | - | 0.2936 | - | - | - | - |
| 15 | 0.0283 | < 0.0001 | 0.0003 | 0.3305 | 0.3570 | 0.0010 |
| 18 | 0.0649 | 0.0190 | 0.0039 | 0.0003 | 0.5784 | 0.0001 |
| 19 | - | 0.2936 | - | - | - | - |

*Only shows Kruskal-Wallis test results when there at least two kmers with length k in a genotype.

**Table S3**. Kruskal-Wallis test of the 31 kmers with mutation rate estimates across all six genotypes of *D. magna* in this study.

| **kmer** | **k** | **Mean copy number** | **Mean per copy mutation rate** | **P-value** |
| --- | --- | --- | --- | --- |
| A | 1 | 22123 | 0.0213 | 0.0116 |
| C | 1 | 5001 | 0.0374 | 0.0002 |
| AC | 2 | 716 | -0.0043 | 0.0618 |
| AG | 2 | 3235 | -0.0179 | 0.0298 |
| AT | 2 | 120 | -0.0239 | 0.0609 |
| AAC | 3 | 337 | 0.0028 | 0.0497 |
| AAG | 3 | 5132 | -0.0281 | 0.0114 |
| AAT | 3 | 137 | -0.0194 | 0.0322 |
| ACG | 3 | 48 | 0.0095 | < 0.0001 |
| AGC | 3 | 190 | 0.0108 | 0.0041 |
| ATC | 3 | 21 | -0.0206 | 0.0672 |
| AAAG | 4 | 82 | 0.0197 | 0.0358 |
| AAAT | 4 | 19 | -0.0029 | 0.1654 |
| AGAT | 4 | 13 | 0.0023 | 0.0001 |
| AAAAC | 5 | 923 | -0.0109 | 0.0039 |
| AACCT | 5 | 7206 | -0.0066 | 0.0167 |
| AAGAT | 5 | 19 | -0.0064 | 0.0011 |
| ACTAT | 5 | 1827 | -0.0017 | 0.0161 |
| AACAGG | 6 | 121 | -0.0013 | < 0.0001 |
| AACTAC | 6 | 95 | -0.0243 | 0.6727 |
| AAGGCG | 6 | 12 | 0.0077 | 0.0479 |
| ATCGCC | 6 | 67 | 0.0016 | < 0.0001 |
| ATATCCC | 7 | 72 | 0.0016 | < 0.0001 |
| AACTGCATC | 9 | 24 | -0.0066 | < 0.0001 |
| AAATAATAAT | 10 | 9 | -0.0367 | 0.0287 |
| AAGGAGGTAG | 10 | 13 | 0.0216 | 0.0251 |
| AAGACTGACTG | 11 | 49 | -0.0253 | 0.0224 |
| ACCACTACTCCG | 12 | 10 | 0.0263 | 0.0364 |
| AACTACTATATAG | 13 | 42 | 0.0057 | 0.0002 |
| ACCAGCCTACCCCGC | 15 | 29 | 0.0016 | 0.0055 |
| ACATCGTCCACGGATCCG | 18 | 8 | 0.0032 | < 0.0001 |

*’Mean copy number’ represents the mean copy number of the kmer across all SC, EC and MA lines lines. ‘P-value, represents the p-value from performing the Kruskal-Wallis test.

**Table S4**. Statistics for the kmers with ten highest and ten lowest |u_j_| for each of six genotypes of *D. magna* collected from three locations, Finland (F), Germany (G) and Israel (I).

| **Genotype** | **Category** | **Mean k** | **Mean GC content** | **Mean \|u_j_\|** |
| --- | --- | --- | --- | --- |
| FA | Highest | 7.6 | 0.33 | 0.071 |
| FA | Lowest | 10.4 | 0.46 | 0.013 |
| FC | Highest | 8.1 | 0.31 | 0.056 |
| FC | Lowest | 9.4 | 0.49 | 0.012 |
| GA | Highest | 7.1 | 0.30 | 0.064 |
| GA | Lowest | 12.5 | 0.57 | 0.015 |
| GC | Highest | 6.7 | 0.34 | 0.055 |
| GC | Lowest | 8.7 | 0.43 | 0.012 |
| IA | Highest | 7.8 | 0.36 | 0.050 |
| IA | Lowest | 7.3 | 0.45 | 0.009 |
| IC | Highest | 8.0 | 0.38 | 0.044 |
| IC | Lowest | 11.4 | 0.49 | 0.010 |

*k represents the kmer length, GC represents the proportion of base pairs that are GC in the kmer

**Table S5**. Two-way ANOVA results testing the relationship between genotype and absolute per copy mutation rate category (high vs low) and GC-content of kmers in *D. magna*.

| **Factor** | **D.f.** | **SumSq** | **MeanSq** | **F-value** | **P-value** |
| --- | --- | --- | --- | --- | --- |
| Genotype | 5 | 0.043 | 0.0085 | 0.202 | 0.9612 |
| Category | 1 | 0.641 | 0.6405 | 15.177 | 0.0002 |
| Genotype:Category | 5 | 0.115 | 0.0231 | 0.547 | 0.7405 |
| Residuals | 108 | 4.558 | 0.0422 |  |  |

*Category high and low represents kmers with the 10 highest and 10 lowest absolute per copy mutation rate, |u_j_|, respectively.

**Table S6**. Pairwise correlations of per copy mutation rates among the 31 kmers shared across the six genotypes of *D. magna* collected from three locations, Finland (F), Germany (G) and Israel (I).

|  | **FA** | **FC** | **GA** | **GC** | **IA** | **IC** |
| --- | --- | --- | --- | --- | --- | --- |
| **FA** | 1 | 0.29 | 0.00 | 0.17 | -0.13 | 0.08 |
| **FC** |  | 1 | 0.45 | 0.15 | 0.41 | 0.43 |
| **GA** |  |  | 1 | -0.15 | 0.60 | 0.63 |
| **GC** |  |  |  | 1 | -0.07 | 0.01 |
| **IA** |  |  |  |  | 1 | 0.67 |
| **IC** |  |  |  |  |  | 1 |

**Table S7.** Total number of reads for each line sequenced in this study.

| **Line** | **Num. of reads** |  | **Line** | **Num. of reads** |  | **Line** | **Num. of reads** |
| --- | --- | --- | --- | --- | --- | --- | --- |
| FA10 | 117276453 |  | GA10 | 67872281 |  | IA10 | 77469618 |
| FA12 | 111118502 |  | GA2 | 74933433 |  | IA1 | 76339146 |
| FA13 | 98944469 |  | GA3 | 78929079 |  | IA2 | 82762562 |
| FA14 | 118671293 |  | GA4 | 78740281 |  | IA4 | 94985444 |
| FA1 | 100759904 |  | GA5 | 79320460 |  | IA5 | 110594565 |
| FA5 | 108817148 |  | GA6 | 71148348 |  | IA6 | 100880878 |
| FA6 | 92034278 |  | GA7 | 76557733 |  | IA7 | 78383275 |
| FASC | 88590180 |  | GA8 | 58644700 |  | IA8 | 90086901 |
| FC12 | 68037364 |  | GASC | 72730208 |  | IASC | 76126994 |
| FC1 | 67658931 |  | GC10 | 75779178 |  | IC10 | 83523929 |
| FC2 | 70000714 |  | GC2 | 96690080 |  | IC1 | 84683921 |
| FC3 | 87568787 |  | GC3 | 74165418 |  | IC3 | 79042669 |
| FC4 | 72157642 |  | GC4 | 100073584 |  | IC4 | 89897718 |
| FC6 | 70810578 |  | GC5 | 107328336 |  | IC5 | 64317095 |
| FC7 | 82224489 |  | GC6 | 92860991 |  | IC6 | 67608868 |
| FC8 | 71891429 |  | GC8 | 106121415 |  | IC7 | 67236965 |
| FCSC | 93661924 |  | GC9 | 105658620 |  | IC9 | 70519081 |
| FAEC1 | 92815980 |  | GCSC | 71010993 |  | ICSC | 64756748 |
| FAEC2 | 101739760 |  | GAEC1 | 83641911 |  | IAEC1 | 77480400 |
| FCEC1 | 79655226 |  | GAEC2 | 70749011 |  | IAEC2 | 84786933 |
| FCEC2 | 92606026 |  | GCEC1 | 119412606 |  | ICEC1 | 89041032 |
|  |  |  | GCEC2 | 107606136 |  | ICEC2 | 89387923 |

**Table S8. Total kmer base pairs in the IA genotype for kmers with unit length less than 10 bp from the t10 and t50 analysis**

| **Line** | **t10** | **t50** | **% Diff** |
| --- | --- | --- | --- |
| IASC | 136558 | 95373 | 43.2 |
| IA1 | 132932 | 91570 | 45.2 |
| IA2 | 158535 | 113486 | 39.7 |
| IA4 | 145021 | 104840 | 38.3 |
| IA5 | 146212 | 103897 | 40.7 |
| IA6 | 163316 | 118659 | 37.6 |
| IA7 | 168011 | 120474 | 39.5 |
| IA8 | 140122 | 98034 | 42.9 |
| IA10 | 151212 | 105073 | 43.9 |

Only kmers with unit lengths k < 10 and are present in both the t10 and t50 analyses for genotype IA are shown. As expected, lowering the tandem repeat length threshold from 50bp to 10bp increased the number of microsatellite loci we were able to detect, which is reflected as an overall increase in kmer base pairs. On average, the total kmer base pairs composed of kmers with k < 10 is 41% larger in the t10 analysis than the t50 analysis. ‘% Diff’ is calculated as the kmer base pairs in t10 minus the total kmer base pairs in t50 divided by that in t50.

**
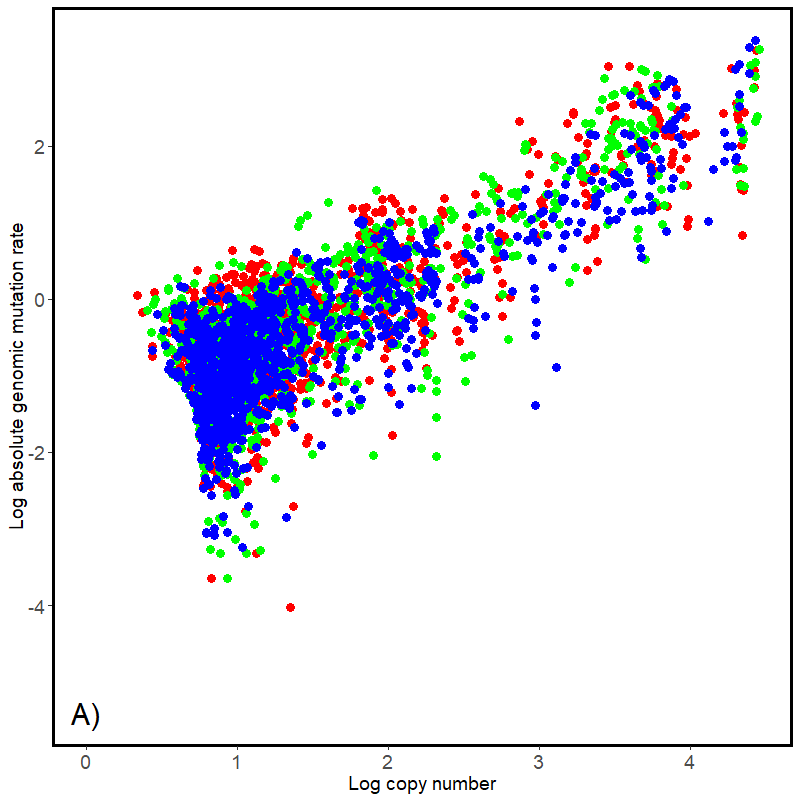

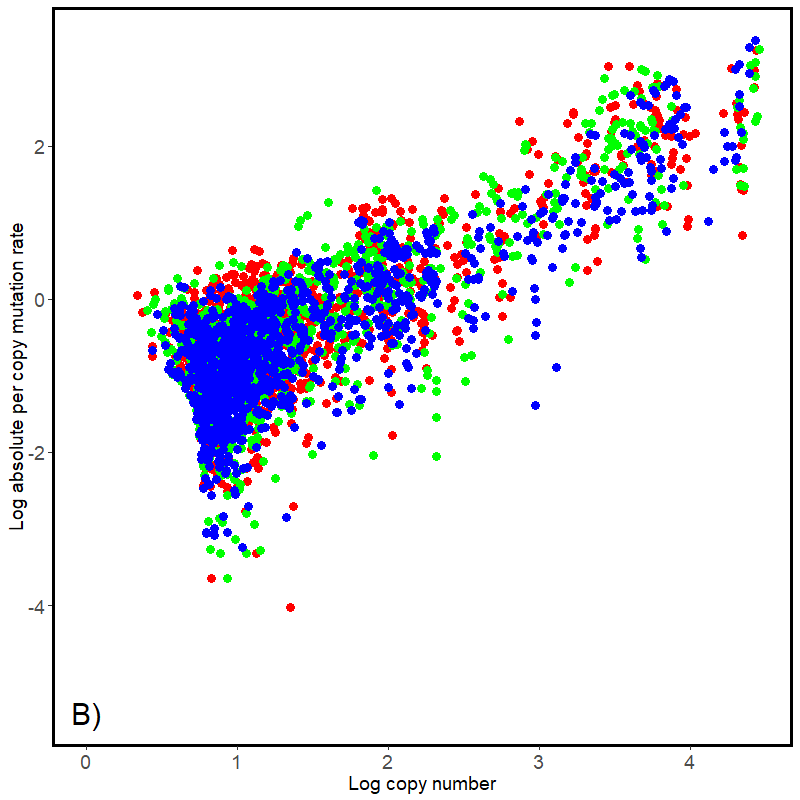
**

**Figure S1**. Absolute genomic mutation rate **(A)** and absolute per copy mutation rate **(B)** plotted against initial copy number for each kmer from each genotype. Red, green and blue represents genotypes from Finland, Germany and Israel, respectively.

**Figure S2**. Total base pairs composed of kmers for SC and MA lines of each genotype. Total base pairs of MA lines represented by the boxplots; white circles represent lines outside 1.5 times of the interquartile range. Total base pairs for SC lines represented by the black squares. Data shown for each of six genotypes of *D. magna* collected from three locations, Finland (F), Germany (G) and Israel (I).


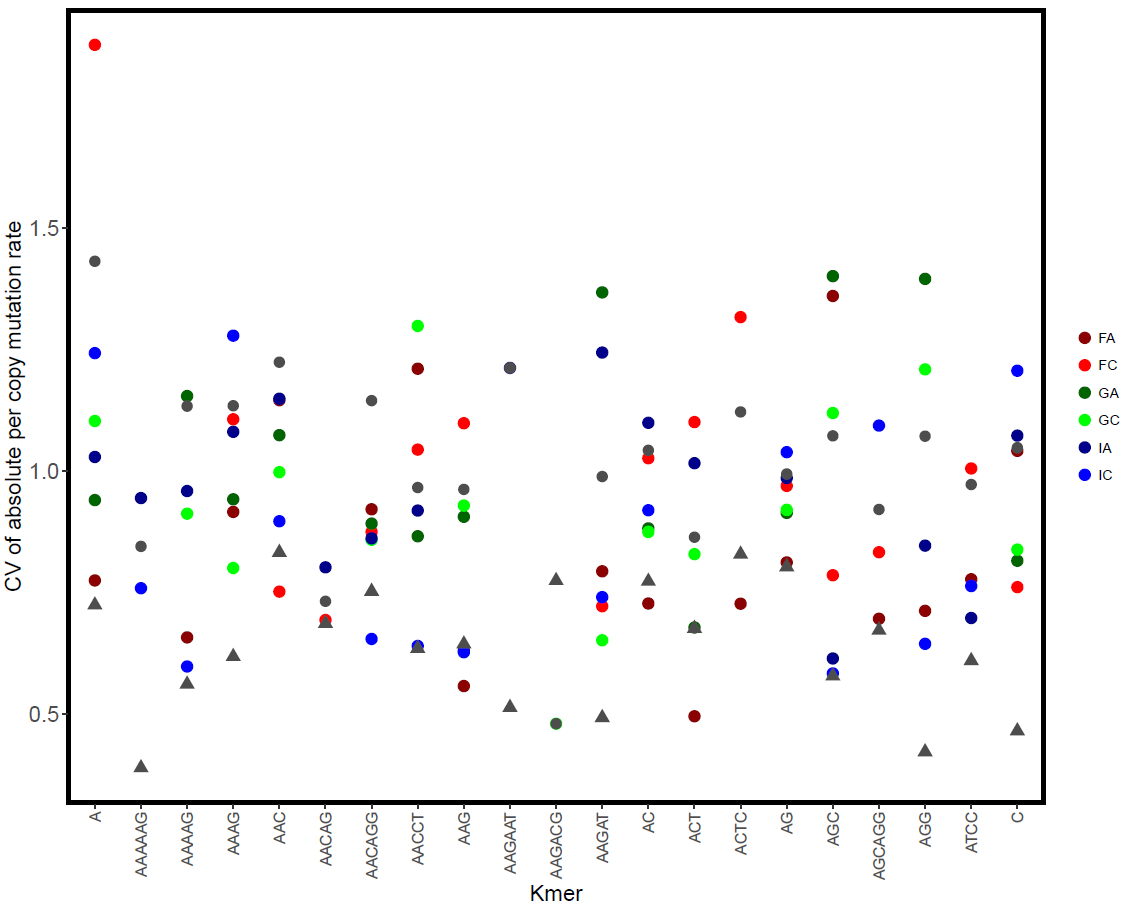


**Figure S3**. Coefficient of variation in |u_i,j_| for each *D. magn*a genotype (circle) and for *D. pulex* (grey triangle) for the 21 kmers shared across species. Grey circles represent the coefficient of variation across all six genotypes of *D. magna* collected from three locations, Finland (F), Germany (G) and Israel (I).


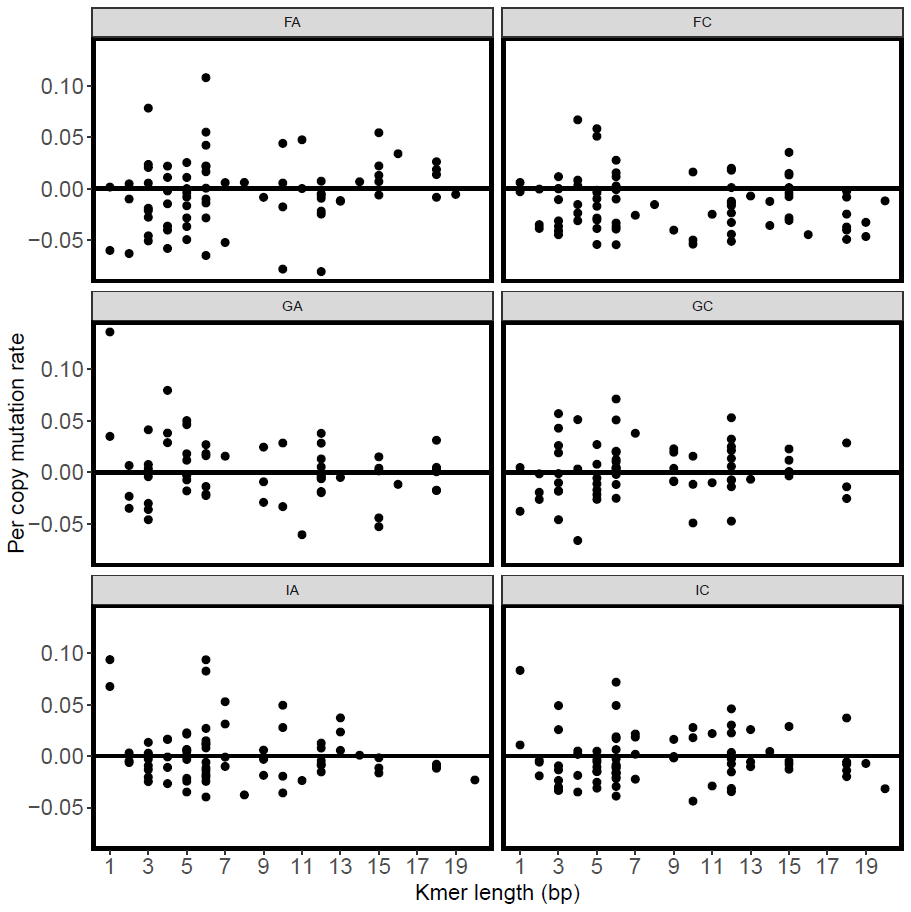


**Figure S4**. Per copy mutation rate of kmers j (u_j_) plotted against kmer lengths for six genotypes of *D. magna* collected from three locations, Finland (F), Germany (G) and Israel (I).


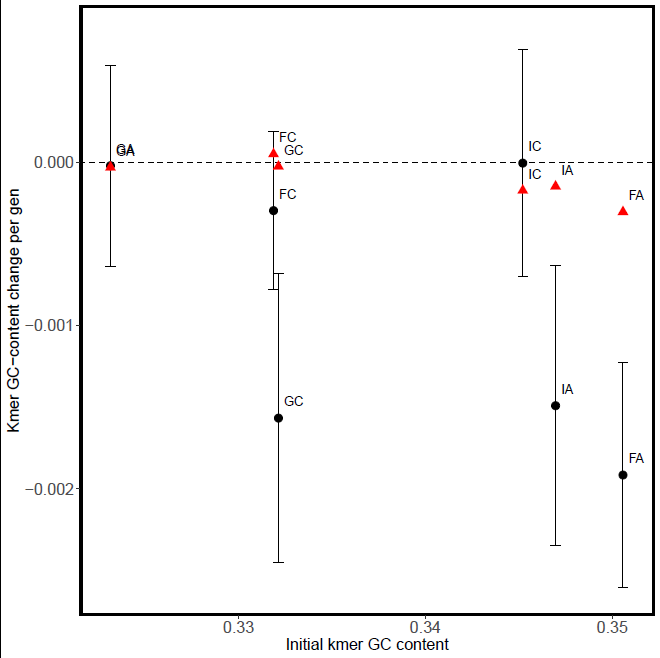


**Figure S5**. Mean (+/- SE) kmer GC-content change plotted against initial kmer GC-content for all six genotypes of *D. magna* collected from three locations, Finland (F), Germany (G) and Israel (I). Black circles and red triangles represent MA and EC lines, respectively.

**
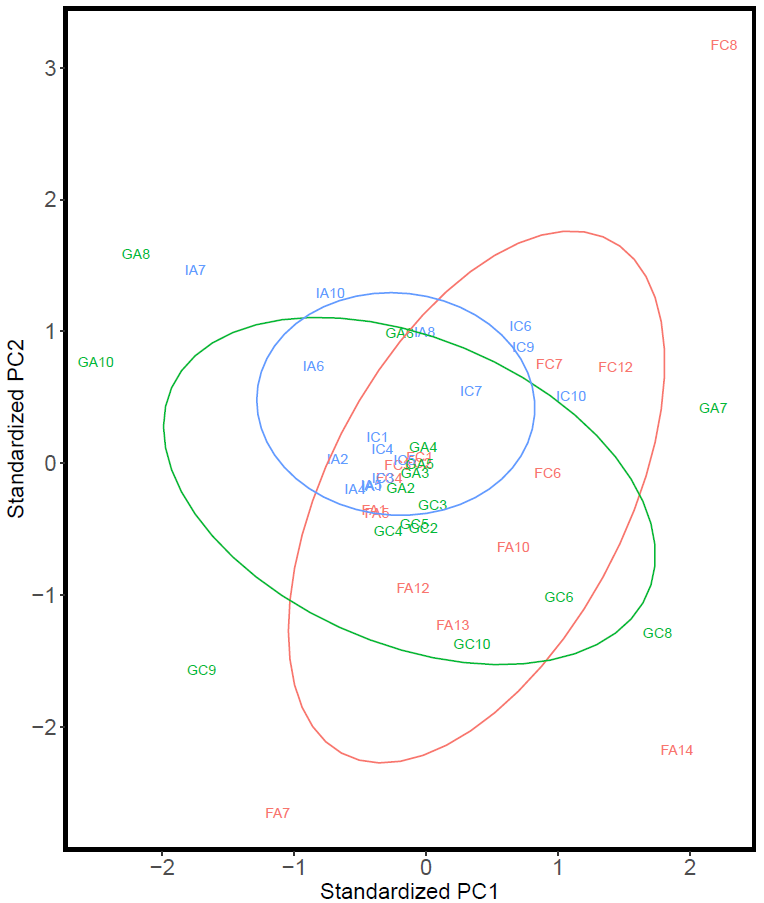
**

**Figure S6**. Population structure using u_j_ for the 31 kmers with mutation rate estimates for all six genotypes of *D. magna* collected from three locations, Finland (F; red), Germany (G; green) and Israel (I; blue). Each MA line is plotted based on the first and second principal components axis.

**
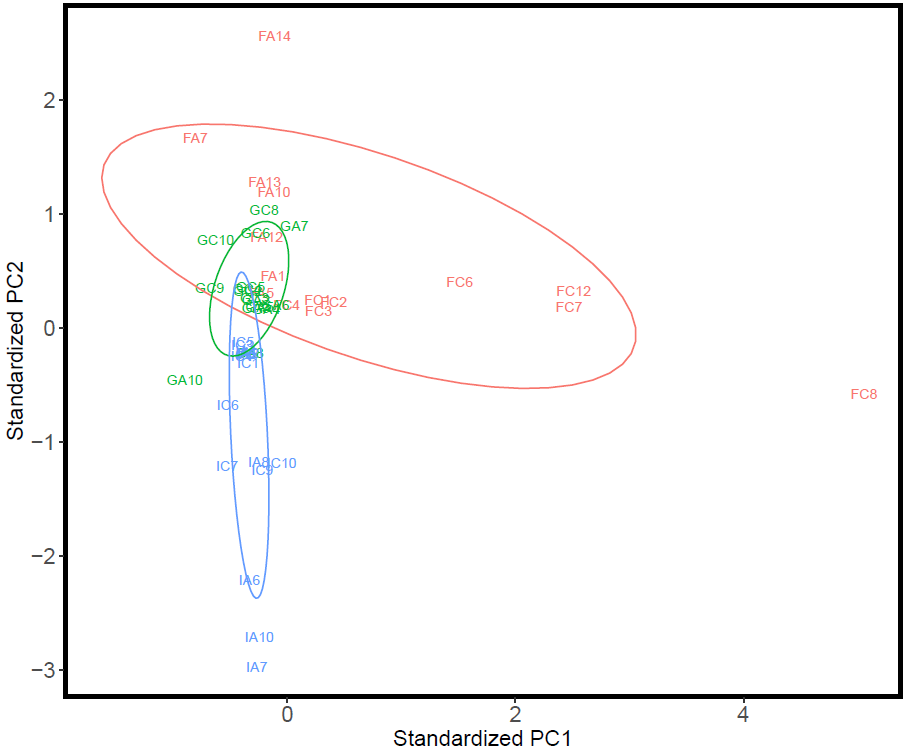
**

**Figure S7**. Population structure using u_j_ for all 144 kmers with mutation rate estimates for all six genotypes of *D. magna* collected from three locations, Finland (F; red), Germany (G; green) and Israel (I; blue). Each MA line is plotted based on the first and second principal components axis. If there was no mutation rate estimate for a kmer in a particular genotype, we set u_j_ as 0. Each MA line is plotted based on the first and second principle components axis.

**Figure S8**. Change in total kmer content (bp) per generation for *D. magna* EC lines, *D. magna* MA lines and *D. pulex* MA lines.

**Figure S9.** **(A)** Estimates of per copy mutations rates for the IA genotype from the t10 and the t50 analysis. The dashed line represents the 1-to-1 line. The average value of u was 0.0052 per copy per generation for the t10 analysis and 0.0040 per copy per generation for the t50 analysis. Per copy mutation rates were significantly correlated between the two analyses (correlation = 0.92, p < 0.0001) and a paired t-test did not find a significant difference (t=0.97, df=44, p=0.42). **(B)** Difference in per copy mutation rates between the t10 and the t50 analysis (u_difference_ = u_t10_ – u_t50_) plotted against kmer unit length (k). Only the 51 kmers with unit length less than 10 (k < 10) and which are present in both the t10 and t50 analyses are shown. Across kmers, 22 of the kmers had a lower mutation rate in the t10 analysis, while 29 kmers had a higher mutation rate in the t10 analysis. A simple linear model found no significant effect of k on u_difference_ (p = 0.752).

**Figure S10.** Schematic of the experimental design for the mutation accumulation experiment used to generate the tissue sequenced for this study.
